# Elimination of Medical Agents from the Body

**Published:** 1865-12

**Authors:** 


### ELIMINATION OF MEDICAL AGENTS FROM THE BODY.

"MM. Bergeon and Lemaitre have lately made some observations on the elimination of medical substances with the sweat. (*Arch. Génér.*) The patients were placed in a dry chamber, where the temperature could be raised to 60° and 65° Cent. The sweat was then allowed to drain off; and as much as from 40 to 60 *grammes* could be collected in this way. They found that the arsenites of potass and soda were eliminated as such. The arsenite of iron was decomposed, the iron passing away with the urine, and the arsenic as an alkaline with the sweat. The protiodide of mercury was eliminated as a bichloride; traces of mercury were found in the sweat; and the iodine was discovered in the saliva and in the urine. Bichloride of mercury was also found in the sweat and in the urine. Iodide of potassium was found in the saliva and in the urine, but not in the sweat. In two cases of albuminuria, albumen was not found in the sweat. A small quantity of sweat, collected from a diabetic patient, contained a large quantity of sugar."

### DEATH OF WM. LYMAN, M. D., OF ROCKFORD, ILL.

Pursuant to a call, the members of the Medical Profession of the city of Rockford, met on the 21st inst., and unanimously adopted the following preamble and resolutions :

WHEREAS, By the dispensation of Providence, Dr. WM. LYMAN has been called from time to eternity ; therefore,

*Resolved*, That by the death of Dr. Lyman, the Medical Profession has been bereft of one of its most devoted and noble members ; society of a true friend and benefactor ; his family of a loving and large hearted protector ; and we, his professional associates, of a friend, who, by his uniform kindness of heart and geniality of manner, has won our esteem and respect.

*Resolved*, That we extend to the family of the deceased our sympathy for their bereavement, and that we attend the funeral in a body.

*Resolved*, That a copy of these resolutions be transmitted by the Secretary to the bereaved family, and copies for publication to the Chicago MEDICAL JOURNAL, Chicago *Medical Examiner*, and the Rockford *Register*.

HENRY STRONG, M. D., Chairman,
G. W. ROHR, M. D., Secretary.

